# Immunomodulation by Gut Microbiota: Role of Toll-Like Receptor Expressed by T Cells

**DOI:** 10.1155/2014/586939

**Published:** 2014-07-24

**Authors:** Mariagrazia Valentini, Alessia Piermattei, Gabriele Di Sante, Giuseppe Migliara, Giovanni Delogu, Francesco Ria

**Affiliations:** ^1^Institute of General Pathology, Università Cattolica del Sacro Cuore, Largo F. Vito 1, 00168 Rome, Italy; ^2^Institute of Microbiology, Università Cattolica del Sacro Cuore, Largo F. Vito 1, 00168 Rome, Italy

## Abstract

A close relationship exists between gut microbiota and immune responses. An imbalance of this relationship can determine local and systemic immune diseases. In fact the immune system plays an essential role in maintaining the homeostasis with the microbiota that normally resides in the gut, while, at the same time, the gut microbiota influences the immune system, modulating number and function of effector and regulatory T cells. To achieve this aim, mutual regulation between immune system and microbiota is achieved through several mechanisms, including the engagement of toll-like receptors (TLRs), pathogen-specific receptors expressed on numerous cell types. TLRs are able to recognize ligands from commensal or pathogen microbiota to maintain the tolerance or trigger the immune response. In this review, we summarize the latest evidences about the role of TLRs expressed in adaptive T cells, to understand how the immune system promotes intestinal homeostasis, fights invasion by pathogens, and is modulated by the intestinal microbiota.

## 1. TLRs and Microbiota

The gut is the largest defense barrier of our body. More than 60% of immune cells are in the gut mucosa, ready to identify and counteract the presence of potential aggressors and inhibit uncontrolled inflammatory reactions [1, 2].

A further protection is represented by the presence of various populations of microorganisms (each encompassing several strains) that condition both the mucosal immune response and the ability of the host to resist aggressive pathogens' attacks [3, 4].

The human gut microbiota is composed of microorganisms that include bacterial communities, yeasts, and bacteriophages (viruses that control the bacterial community, and in particular, the ability of bacteria to regulate our metabolism) all residing in the intestinal tract. This community encompasses trillions of bacteria with an estimated biomass of 1 kg [5].

Molecular and metagenomic approaches have allowed identifying the main bacterial communities present in the digestive tract, their role in health, and their relationships with specific diseases [6–8].

The gut immune system monitors the communities that flow in the lumen and, in healthy conditions, reacts against potentially pathogenic organisms by inducing inflammation, while maintaining tolerance towards most members of commensal microbiota [9–11].

Therefore, the defense of the organism requires a careful surveillance able to distinguish microbes with pathogenic potential (pathobionts) from nonpathogenic microorganisms (mainly symbionts) [12].

The ability of these cells to discriminate pathogens from commensals is mediated by pattern recognition receptors (PRRs) that include the families of toll-like receptors (TLRs), nucleotide-binding oligomerization domain- (NOD-) like receptors (NLRs), C-type lectin receptors (CLRs), cytosolic DNA receptors (CDRs), and RIG-I-like receptors (RLRs). In particular, TLRs are mostly (but not exclusively) present on the membrane of immune and epithelial cells [13] and NODs are present in the cytoplasm of enteric cells [14]. TLRs and NODs are capable of recognizing conserved molecular motives, generally divided in microbe-associated molecular patterns (MAMPS, expressed by resident microbiota) and pathogens-associated molecular patterns (PAMPS, produced by microbial invaders). Their engagement induces several intracellular signaling cascades resulting in the production of cytokines, chemokines, and transcription factors that are essential for the maintenance of the gut homeostasis and/or infection control [15].

Therefore, TLRs play an important role in suppressing the activation of the inflammatory cascade to maintain the balance of intestinal homeostasis and in promoting inflammatory responses to pathogens [16–18].

Eleven different transmembrane proteins belong to the TLR family. Although they are constantly exposed to a significant charge of commensal bacteria, they are able to restrain inflammation in steady-state conditions, keeping a tone of hyporesponsiveness against the intestinal flora [19].

Recent studies suggest that the mechanisms that limit immune activation belong to potential synergistic actions from both host and bacterial effector molecules. Such molecules are able to antagonize and modulate the signal transmission mediated by TLRs, acting along the signal transduction from the TLRs or at the level of production of effector molecules [20].

TLR2 is involved in the recognition of Gram-negative and Gram-positive bacteria and yeast. At the same time, different evidence proves that TLR2 is able to switch its ability to produce pro- and anti-inflammatory responses by dimerization with several coreceptors such as TLR2 itself, TLR1, TLR6, and TLR10 [21]. Recent studies suggest that TLR2/TLR6 dimerization activates the TLR2-MyD88-IRAK-TRAF-NIK-IKK-NF-*κ*B signal transduction pathway that induces transcription of proinflammatory molecules, while TLR2/TLR1 dimerization promotes the anti-inflammatory pathway that leads to the expression of IL-10 and the transdifferentiation of Th17 and iTreg cells [22].

In order to maintain the immune homeostasis, the host uses several mechanisms that limit and inhibit the inflammatory responses mediated by TLR2. One of these is the modulation of TLR2 signaling through the expression of negative regulators such as the toll-interacting protein (TOLLIP). TOLLIP inhibits IRAK binding TLR2 or TLR4, thereby breaking down this proinflammatory pathway [23]. In addition, commensal bacteria provide other supplementary mechanisms through which they prevent gut colonization by pathogens, as exemplified by the action of* Bacteroides fragilis* through its unique surface polysaccharide (PSA) [24].

TLR4 is expressed at low levels on the surface of epithelial gut cells, where it plays a role in the intestinal mucosal defense against Gram-negative bacteria. TLR4, after activation by lipopolysaccharide (LPS) or endotoxin from Gram-negative bacteria, dimerizes with CD14 and MD-2 and induces the consequent signaling cascade that ultimately leads to the activation of a proinflammatory response. TLR4 signaling is regulated by the expression of the transmembrane protein ST2. ST2 sequesters MyD88 and TIRAP (adaptor proteins associated with TLR), thus antagonizing TLR4 functions and contributing to the persistence of the hyporesponsiveness to commensal microbial community [25, 26].

TLR5 is the innate immune receptor for bacterial flagellin. As the other TLRs, TLR5 is involved both in the recruitment of the adaptor MyD88, upregulating a signaling cascade of proinflammatory transcription factors, and in the maintenance of the gut microbiota homeostasis. Indeed, many commensal species that colonize the gut express flagellin. Activation of TLR5 signaling displays a proinflammatory effect by regulating the production of IL-17 and IL-22 that in turn promote antimicrobial defense essential for clearance of pathogens and protective effects. On the other side, the interaction between Tlr5 and flagellin also leads to the expression of antiapoptotic genes that are correlated with the protective effect of the receptor against normal commensal such as* E. coli* [27].

A large body of evidence shows that TLR9 engagement has contrasting effects on activation of nuclear factor-*κ*B, depending on its expression on apical or basolateral surface of intestinal epithelial cells (IECs) and thereby playing an important role in the gut epithelial homeostasis. TLR9 recognizes intracellular bacteria, by binding the unmethylated CpG motifs of bacterial DNA. While the interaction with engagement of basolateral Tlr9 has been reported to enhance the activation of NF-*κ*B, binding of CpG with apical Tlr9 seems to promote the ubiquitination of I*κ*B that prevents the activation of NF-*κ*B [28, 29].

## 2. TLRs and Adaptive T Cells: Activation and Functions

Naïve CD4^+^ T cells migrate from the thymus to periphery, under environmental signals that induce their maturation and functions. Depending on microbial and host signals, T cells differentiate into pro- and anti-inflammatory subsets, such as Th1, Th2, Th17, and iTreg.

The presence of Th17 and iTreg cells in the healthy gut has been largely demonstrated. Th17 cells are a specific lineage of CD4^+^ Th cells that produce inflammatory cytokines such as IL-17a, IL-17f, IL-21, and IL-22 [30, 31]. They promote the host defense against fungal and bacterial infections, such as* Candida albicans*,* Pseudomonas aeruginosa*,* Klebsiella pneumonia*,* Streptococcus pneumonia,* and* Citrobacter rodentium* [32, 33]. Differentiation of CD4^+^ T cells into Th17 in the gut depends on the stimulation by intestinal microbiota and their products, such as serum amyloid A (SAA), from segmented filamentous bacteria and extracellular ATP [34].

iTreg cells, also defined as inducible suppressor cells, are a subset of CD4^+^ Th cells that express CD4, CD25, and Foxp3 (Forkhead Box 3, the nuclear transcription factor specifically involved in Treg differentiation) [35].

iTreg are capable of suppressing the activation of the immune system, regulating the homeostasis and tolerance to self-antigens. Several recent studies have demonstrated the presence of Treg cells that secrete IL-10, an anti-inflammatory cytokine [36]. These Treg subsets are not found in thymic environment but are present in peripheral tissues, as the gut [37].

Albeit activation of TLRs is the hallmark of the innate immune response, it has been demonstrated that TLRs are also important for adaptive immune cell function as regulation of B lymphocytes development [38] and antibody production [39]. It has also been demonstrated that certain TLRs are also expressed on T lymphocytes [40] and that TLRs ligands can modulate directly functions of T cell such as signaling in Treg cells [41] or development and effector functions of the various subsets of T helper cell [42].

### 2.1. TLR2 and T Cells

TLR2 is able to trigger proliferation and cytokine production (in particular IL-2 and IFN-*γ*) of effector T cells activated via TCR [43], thus regulating the host's immune system against pathogens. Mokuno et al. also reported that stimulation of TLR2 on *γ* *δ* T cells increases significantly their proliferative response [44]. In CD8^+^ cells, TLR2 induces T-bet activity, IFN-*γ* [45], TNF-*α*, and other cytotoxic mediators [46, 47]. The same effects have been observed in natural killer T cells (NKT), where the stimulation of TLR2 enhances the expression of Fas-L [48]. Recently, the literature has illustrated the important role of TLR2 in T helper subsets for proliferation and survival [49], cell migration [50, 51], protection against tuberculosis and filarial infections [52, 53], and reduction of IL-4 production [54].

Tlr2 enhances also IL17 productions in CD4^+^ T cells, promoting experimental autoimmune encephalomyelitis (EAE) pathogenesis and severity [55]. We observed that a polymorphism of Tlr2 modulates severity, remission, and lesion distribution during EAE, although it does not influence disease incidence (manuscript in preparation). An interesting point is that Tlr2 stimulation promotes the differentiation of iTregs into a Th17 [56], which may enhance microbial clearance but may also increase the risk of autoimmune reactions. This role of Tlr2 may be relevant in the pathogenesis of MS (and of its experimental model, the EAE), since iTregs protect from autoimmune aggressions, whereas Th17 cells expand in the periphery and accumulate in the CNS, where they support demyelination [22, 57].

It has been shown in human T cells [56] and we are confirming it in experimental models (manuscript in preparation) that engagement of TLR2 expressed on T cells modulates Fox-P3 mRNA, in a strain-dependent and activation status-dependent manner. These observations imply that products derived from microbiota or pathobiota can modulate directly T cell polarization, in addition to their mobility. Thus, we suggest that environmental infectious agents (mainly viruses and bacteria) can influence autoimmune diseases in terms of lesions distribution and severity of disease along a pathway that, through engagement of TLRs, involves CD44, its ligands, and T cell functions.

### 2.2. TLR3 and T Cells

TLR3 recognizes viral components and double-stranded RNA (dsRNA) generated as an intermediate during viral replication. One of the main consequences of its induced-signaling in innate immune cells is the secretion of massive amounts of type I IFNs which play an antiviral role. TLR3 localization in immune cells, including resting T lymphocytes, is mainly intracellular and is capable of recognizing phagocytosed foreign nucleic acids from extracellular space; however, it has been detected at the cell surface of T cells following activation [58], similarly to what happens to TLR2 after stimulation with anti-CD3 antibodies.

The mRNA specific for TLR3 has been found in human CD8^+^ T cells [59], in both effector memory and effector cells, but not in naïve or central memory cells. Its expression did not affect the cytolytic activity but could costimulate CD8^+^ T cells, increasing IFN-*γ* secretion; for example, it has been described also for TLR2 in CD4^+^ T cells [60].

### 2.3. TLR4 and T Cells

Tlr4 promotes EAE and arthritis by increasing the secretion of IFN-*γ* and IL-17 [49, 61], but it has been shown to decrease IFN-*γ* and IL-17 in experimental colitis [62].

Similar to Tlr2, Tlr4 enhances the severity of autoimmune disorders (EAE) in mice, where it promotes IFN-*γ* and IL-17 production by *γ* *δ* T cells [63] and IL-2 secretion and proliferation of NKT [64]. However, while the role of Tlr4 in triggering autoimmune diseases is well established, its influence in cytokine production is still debated.

Cell trafficking plays a fundamental role in autoimmune diseases. It has been demonstrated that Tlr4 is directly involved in cell migration by its ability to bind fibronectin [65].

### 2.4. TLR9 and T Cells

TLR9 engagement is important for T cell survival by decreasing apoptosis, promoting entrance in cell cycle, and arresting the rate of dsDNA break repair, as showed in a study about radiotherapy [66]. It has been demonstrated that oligodeoxynucleotides containing CpG motifs (CpG-ODN) cause a costimulation of T cells similar to that obtained by stimulation of CD28, independent of APC. This intrinsic effect of CpG-ODN via Tlr9 on T cells may explain, at least in part, the powerful adjuvanticity of bacterial DNA and of CpG-ODN on antigen-specific T cell responses in vivo and the efficacy of DNA-based vaccines possessing immunostimulatory sequences [67].

## 3. Microbiota, TLR, and T Cell Modulation

Several studies have shown that individual species of the microbiota modulate the ratio among the different types of immune cells, such as Th17 cells and Foxp3^+^ regulatory T cells, suggesting that the composition of the microbiota may have an important influence on the immune response. Numerous reports have shown that alterations in gut microbiota can induce the activation of effector T cells over iTregs and, consequently, trigger the development of autoimmune/inflammatory diseases [68]. These studies identified specific gut commensals that are able to induce either Th17 or Treg responses that are, respectively, associated with development or protection from disease [69].

It has been shown that mice lacking components of the TLR signaling machinery, as Tlr2, Tlr4, or MyD88, are highly susceptible to dextran sodium sulfate- (DSS-) induced intestinal inflammation [70, 71].

*Microbiota, TLRs, and Tregs*. In steady state, the gut is a rich source of TLR ligands from commensal bacteria, some of which have been recently associated with diseases in mouse models of colitis and in human inflammatory bowel diseases. One key antigen that drives gut pathology is flagellin, the major structural subunit of bacterial* flagella* [72]. Flagellin appears to play a central role in the balance and function of T-effector and iTreg cells [73]. In fact, flagellin acts as a TLR5 ligand on CD4^+^ T cells. Low concentrations of flagellin enhance the expression of Foxp3 and the consequent suppressive effect of Treg, whereas high concentrations stimulate T-effector Tlr cell function.

Several studies on mouse models show that TLR2 is involved in regulatory immune responses in the gut. Minimal disruption of the epithelial barrier, resulting from the administration of ethanol or of AT1002 (*V. cholera zona occludens* toxin hexapeptide), leads to IL-10 secretion in addition to the induction of persistent CD4^+^ LAP^+^ (latent TGF-*β*-associated with latency-associated peptide) cells [74]. The mechanism of induction of these cells is not yet clear, but it has been demonstrated to depend on the presence of an intact intestinal flora which acts, at least in part, via Tlr2 stimulation of* lamina propria* CD11c^+^ DCs. Thus, it is likely that the activation of these cells promotes the maintenance of homeostasis against possible intestinal bacterial invasion, before Foxp3^+^ iTreg cells reactions [75].

*Bacteroides fragilis*, a common member of the microbiota, prevents trinitrobenzene sulfonic acid- (TNBS-) induced colitis in mice by producing PSA (capsular polysaccharide A). PSA enhances Treg function via Tlr2 signaling directly in iTregs, promoting tolerance [76]. Administration of PSA prevents or reduces the severity of disease in model of TNBS-induced colitis, and Tlr2^−/−^ animals treated orally with PSA are not protected from colitis [37, 77].* B. fragilis* can also release PSA in outer membrane vesicles (OMVs) sensed by DC via Tlr2, inducing growth arrest and the production of DNA-damage-inducible protein (Gadd45a) in DC, and an increase in IL-10 production from Foxp3^+^ iTreg cells [78]. IL-10, in turn, is required for the induction of homeostasis of effector T cell, since blocking the IL-10 receptor during colonization results in immune deviation [79].

Binding of TLR9 to DNA derived from the microbiota plays a critical role in iTreg/T-effector cells balance and in host defense against* Encephalitozoon cuniculi*, a microsporidian parasite that induces diarrheal, respiratory, and neurological diseases in immunocompromised humans [80].

The simultaneous engagement of multiple TLRs by products from microbial communities or invasive pathogens may vary signal strength [81] and effects. An example of this complex mechanism is that components of host's microbiota, once sensed through Tlr2, 4, and 9, activate a protective T cell responses to* Toxoplasma gondii* oral infection [82].

*Microbiota, TLRs, and Th17*. Despite the large body of works, the role of TLRs in the modulation of the adaptive Th17 cells in the gut is not unequivocal. It has been shown that TLR9-deficient mice have decreased numbers of* lamina propria* Th17 cells [80] and that the differentiation of intestinal Th17 cells is enhanced in vitro by the addition of flagellin, a Tlr5 ligand [83]. These results suggest a potential role for TLR5-dependent signaling also in Th17 differentiation. In contrast, MyD88 and TIR domain-containing adaptor inducing IFN-*β* (Trif) double deficient mice have normal numbers of LP Th17 cells in the small and large intestines [34, 84]. Thus, further studies are needed to clarify the role of TLRs in the induction of intestinal Th17 cells, in which other molecules signaling through MyD88 or Trif may play a role opposite to that of Tlr9 [85].

In addition to TLR ligands, intestinal bacteria have been shown to provide large amounts of extracellular ATP [12] that is a critical factor produced by intestinal commensal bacteria for the induction of the Th17 phenotype. It has been reported that the addition of the supernatant from intestinal commensal bacteria promotes the polarization of naïve Th cells into Th17 that is severely inhibited by the presence of the ATP degrading enzyme [34].

The presence of segmented filamentous bacteria (SFB) in the murine gut, for example, is associated with induction of Th17-mediated autoimmune/inflammatory diseases such as colitis, arthritis, and EAE [86, 87]. The mechanisms through which SFB-derived molecules induce IgA production and Th17 differentiation are still unknown. It is also unclear if SFB directly activate T and B cells or rather influence other intestinal cells, such as epithelial cells or DC. SFB protect from invasion by the pathogenic microorganism* Citrobacter rodentium* by inducing IL-22 production by Th17 cells that inhibits the growth of this microorganism [88]. Similarly, SFB protect from development of type 1-diabetes (T1D) the nonobese diabetes (NOD) mice [89], a spontaneous model of T1D, in an IL-17-dependent manner.

## 4. Conclusions

**Figure 1:**
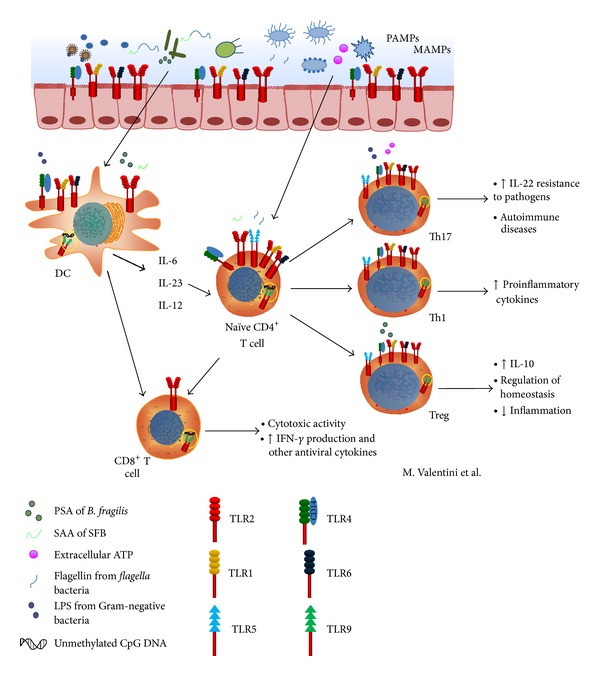
The mammalian gut microbiota is involved in the intestinal homeostasis and shapes the adaptive immune system. The interaction between TLRs and different ligands (such as polysaccharide A of* B. fragilis*, serum amyloid A protein of segmented filamentous bacteria, extracellular ATP from intestinal microbiota, flagellin, LPS, and unmethylated CpG of bacterial DNA) induces CD8^+^ T activation and naïve CD4^+^ T polarization towards Th17, Th1, and Treg subsets. The Th17 cells act against pathogens and promote autoimmune disease. The Th1 cells upregulate the production of proinflammatory cytokines, whereas Treg cells produce IL-10 and are involved in the maintenance of homeostasis and in a downregulation of inflammation. Moreover, CD8^+^ T cells induce the IFN-*γ* and other cytotoxic mediators production.

The role of microbiota in the activation and in the modulation of T cells functions is still under scrutiny. The specific mechanisms by which commensals trigger or hamper immune responses and immune-mediated diseases are still unknown. As summarized in Figure 1, we focused our attention on the evidence indicating the possibility that microbiota acts through TLRs expressed by adaptive T cells to provide regulatory signals.
